# Regulation of RasGRP1 Function in T Cell Development and Activation by Its Unique Tail Domain

**DOI:** 10.1371/journal.pone.0038796

**Published:** 2012-06-12

**Authors:** Deirdre M. Fuller, Minghua Zhu, Xiaohua Song, Chih-wen Ou-Yang, Sarah A. Sullivan, James C. Stone, Weiguo Zhang

**Affiliations:** 1 Department of Immunology, Duke University Medical Center, Durham, North Carolina, United States of America; 2 Department of Biochemistry, University of Alberta, Edmonton, Alberta, Canada; Beth Israel Deaconess Medical Center, Harvard Medical School, United States of America

## Abstract

The Ras-guanyl nucleotide exchange factor RasGRP1 plays a critical role in T cell receptor-mediated Erk activation. Previous studies have emphasized the importance of RasGRP1 in the positive selection of thymocytes, activation of T cells, and control of autoimmunity. RasGRP1 consists of a number of well-characterized domains, which it shares with its other family members; however, RasGRP1 also contains an ∼200 residue-long tail domain, the function of which is unknown. To elucidate the physiological role of this domain, we generated knock-in mice expressing RasGRP1 without the tail domain. Further analysis of these knock-in mice showed that thymocytes lacking the tail domain of RasGRP1 underwent aberrant thymic selection and, following TCR stimulation, were unable to activate Erk. Furthermore, the deletion of the tail domain led to enhanced CD4^+^ T cell expansion in aged mice, as well as the production of autoantibodies. Mechanistically, the tail-deleted form of RasGRP1 was not able to traffic to the cell membrane following stimulation, indicating a potential reason for its inability to activate Erk. While the DAG-binding C1 domain of RasGRP1 has long been recognized as an important factor mediating Erk activation, we have revealed the physiological relevance of the tail domain in RasGRP1 function and control of Erk signaling.

## Introduction

The Ras family proteins are critical components of many signaling pathways and serve to regulate a variety of cell functions, such as proliferation and differentiation. The most well-characterized signaling pathway regulated by Ras is the Erk (extracellular signal-regulated kinases)-MAPK (mitogen activated protein kinase) pathway. Upon activation, Ras recruits Raf-1, a serine/threonine kinase, to phosphorylate MEK (MAPK/Erk kinase), which in turn activates Erk1 and Erk2. In T cells, Ras is activated by two families of Ras GEFs (guanine exchange factors), RasGRP (Ras guanyl releasing protein) and Sos (son of sevenless). It was recently shown that Sos contains an allosteric Ras-GTP binding pocket that binds to Ras-GTP and enhances Sos activity, suggesting that RasGRP1 may be necessary to prime Sos [1]. Studies analyzing mice deficient in either RasGRP1 or Sos1 indicate that both GEFs are critical in thymocyte development [2], [3]. These proteins may have disparate roles in the different stages of thymocyte development as the ratio of Sos1 to RasGRP1 expression changes dramatically upon the DN3 to DP transition [3].

RasGRP1 is most highly expressed in T cells but is also detected in B cells, neuronal cells, and mast cells [4], [5]. Like the other members of the RasGRP family, RasGRP1 contains a catalytic region, which consists of a REM (Ras exchange motif) and a CDC25 (cell division cycle 25) domain, a pair of EF hands (calcium binding), and a C1 domain (DAG binding) [6]. Studies examining the structural domains of RasGRP1 have shown that deletion of the C1 domain impairs Ras activation through disruption of RasGRP1-DAG binding [7], [8], [9]. However, RasGRP1 also has a C-terminal tail domain, consisting of about two hundred amino acid residues [6]. Recently, studies have implicated two domains, the PT (plasma membrane targeting) and SuPT (suppressor of PT), located in the C-terminus of RasGRP1 as important mediators of protein localization [10], [11]. However, the physiological relevance of this tail domain and its potential role in TCR-mediated signaling has yet to be discovered.

The importance of RasGRP1 in TCR signaling was first suggested in Jurkat T cells, in which overexpression of RasGRP1 enhances Ras/Erk signaling [4]. Correspondingly, analysis of a RasGRP1-deficient Jurkat line demonstrated that RasGRP1 is indispensable for antigen receptor- and PMA-triggered Erk activation. Furthermore, RasGRP1-dependent Erk activation relies upon its DAG-binding C1 domain [9]. RasGRP1-deficient mice have severely reduced numbers of single positive thymocytes and, consequently, very few mature T cells in the periphery, while B cell development is not affected. Further analysis of RasGRP1^−/−^ thymocytes reveals their inability to activate Erk after stimulation with phorbol 12-myristate 13-acetate (PMA), a DAG-analog [2]. Another study has shown that RasGRP1 is essential for the weak TCR signals necessary for Erk activation during positive selection. However, stronger signals that induce JNK and p38 activation leading to negative selection can be transmitted independently of RasGRP1 [12].

Interestingly, RasGRP1^−/−^ mice develop an autoimmune disorder marked by splenomegaly and auto-antibody production. This disease is primarily mediated by T cells that exhibit several functional defects [13], [14]. Furthermore, this phenotype arises despite reports that regulatory T cell function is intact and may be enhanced in RasGRP1^−/−^ mice [15]. Contrary to these results, Priatel *et* *al.* assert that this autoimmune condition does not develop upon backcrossing RasGRP1^−/−^ mice onto a B6 background [16]. The reason for these disparate results is unknown but could be due to differences in the ages of the mice used or in the environmental conditions in which the mice were housed.

In accordance with the idea that RasGRP1 regulates autoimmunity in mice, a study analyzing a cohort of patients with systemic lupus erythematosus (SLE) discovered 13 new splice variants of RasGRP1 transcripts, resulting in diminished RasGRP1 activity in these patients. Also, two SLE patients have T cells containing very little, if any, RasGRP1 protein, supporting a role for RasGRP1 in the onset of autoimmunity [17]. Another study examining microRNA expression in lupus CD4^+^ T cells reveals that RasGRP1 can be targeted by miR-21, ultimately contributing to the DNA hypomethylation seen in patients with SLE [18]. Moreover, RasGRP1 is one potential protein whose structural mutation or deregulated expression could mimic oncogenic Ras mutations [19], which are detected in many solid tumors and a subset of T lymphocytic leukemia [20], [21], [22]. Therefore, understanding the regulation of RasGRP1 and its role in Erk activation is essential to comprehend the establishment of T cell tolerance, autoimmunity, and transformation.

Knowing the importance of RasGRP1 in T cell signaling and that RasGRP1 contains a unique tail domain, we undertook the task of elucidating the role of this domain in RasGRP1 function. To accomplish this, we generated RasGRP1^d/d^ knock-in mice, which express a form of RasGRP1 lacking the tail domain. While previous studies have highlighted the irreplaceable role of the C1 domain in RasGRP1-mediated Erk activation, our findings reveal a novel means by which the tail domain of RasGRP1 regulates TCR-mediated Ras signaling.

## Results

### Generation of RasGRP1^d/d^ mice

**Figure 1:**
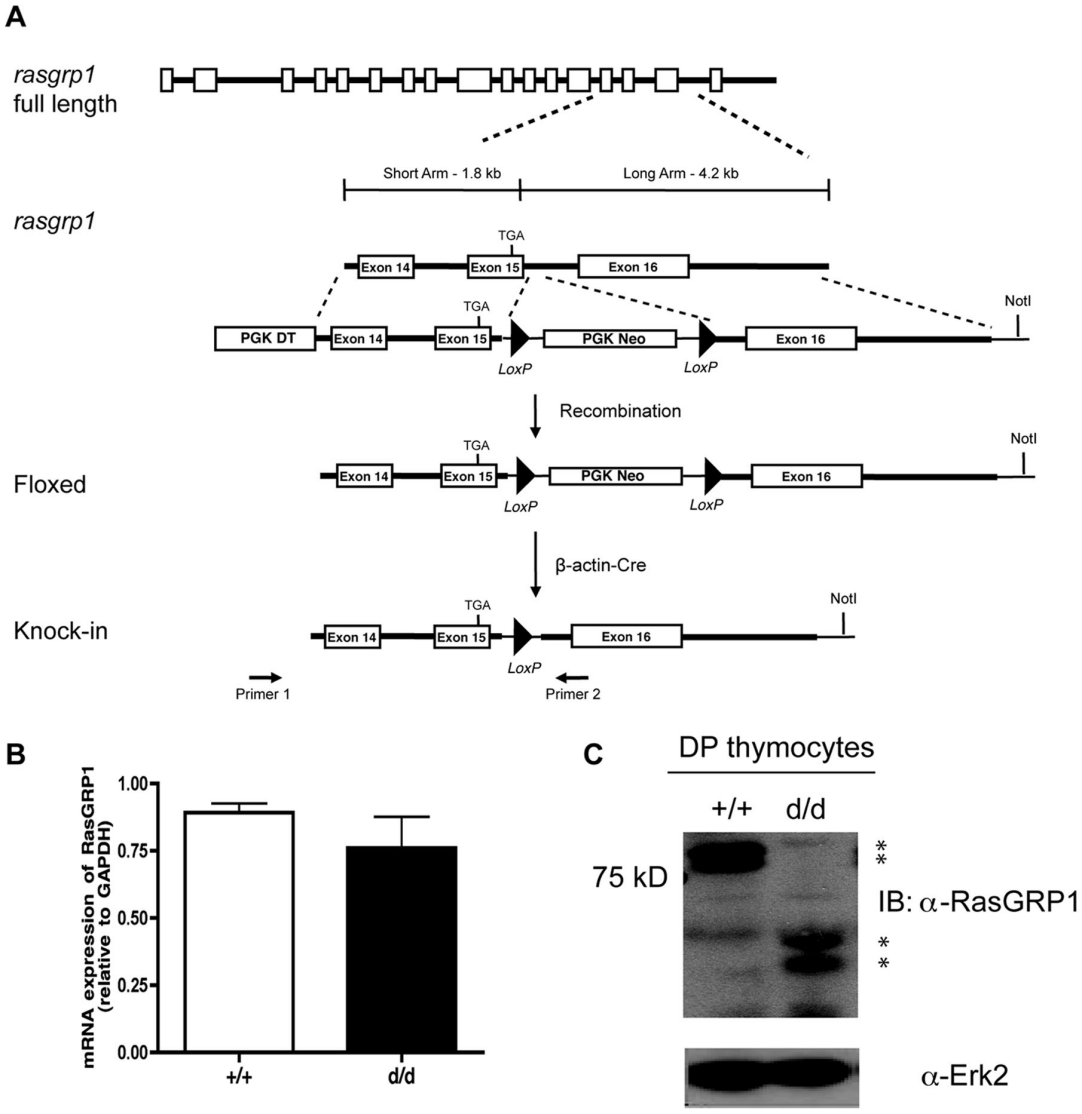
Generation of RasGRP1^d/d^ mice. (A) Targeting construct showing insertion of a stop codon into exon 15 of *rasgrp1*. (B) Real-time PCR analysis of RasGRP1 transcript in DP thymocytes from RasGRP1^+/+^ and RasGRP1^d/d^ mice. (C) Western blot analysis of RasGRP1 expression in DP thymocyte lysate from RasGRP1^+/+^ and RasGRP1^d/d^ mice. * indicates two RasGRP1 protein isoforms on the SDS-PAGE.

To assess the function of the RasGRP1 tail domain, we designed a targeting construct to generate knock-in mice with the tail domain deleted, which we designated as RasGRP1^d/d^ (d=deleted) mice. *Rasgrp1* consists of seventeen exons; we inserted a stop codon, TGA, into exon 15 to ensure retention of the C1 domain but deletion of the tail. This insertion site, in addition to being immediately downstream of a *Bgl II* restriction site, is located between the C1 domain and the SuPT domain. Our targeting vector, as depicted in Figure 1A, was used to transfect ES cells. Positive clones were then used to generate chimeric mice. To remove the PGK-Neo cassette, chimeric mice were crossed with β-actin-Cre transgenic mice to generate RasGRP1^d/+^ mice. These mice were then backcrossed onto a B6 background for ten generations. Intercrossing RasGRP1^d/+^ mice led to RasGRP1^d/d^ offspring at an expected ratio with no visible defects in viability, fertility, or behavior.

Next, we confirmed correct targeting by sequencing a PCR fragment of RasGRP1 amplified from thymocyte cDNA. Sequencing results indicated that a TGA stop codon was correctly inserted towards the 3′ end of exon 15 (data not shown). We also performed real-time PCR analysis to demonstrate comparable transcript expression in RasGRP1^+/+^ and RasGRP1^d/d^ DP thymocytes (Figure 1B). We further analyzed the expression of the truncated RasGRP1 protein by Western blotting to confirm successful gene targeting. Western blot analysis of thymocyte lysates using a monoclonal antibody against the C-terminal portion of RasGRP1 revealed the presence of endogenous RasGRP1 in wildtype but not RasGRP1^d/d^ or RasGRP1^−/−^ lysates (data not shown). Blotting with a polyclonal antibody against the CDC25 domain of RasGRP1 revealed the presence of faster migrating bands in RasGRP1^d/d^ thymocyte lysates than in wildtype samples (Figure 1C). However, expression of mutant RasGRP1 protein was mildly reduced, by ∼30% compared to the wildtype band volume, suggesting that the deletion of the tail domain affects RasGRP1 protein stability.

### Defective thymic and peripheral development in RasGRP1^d/d^ mice

**Figure 2:**
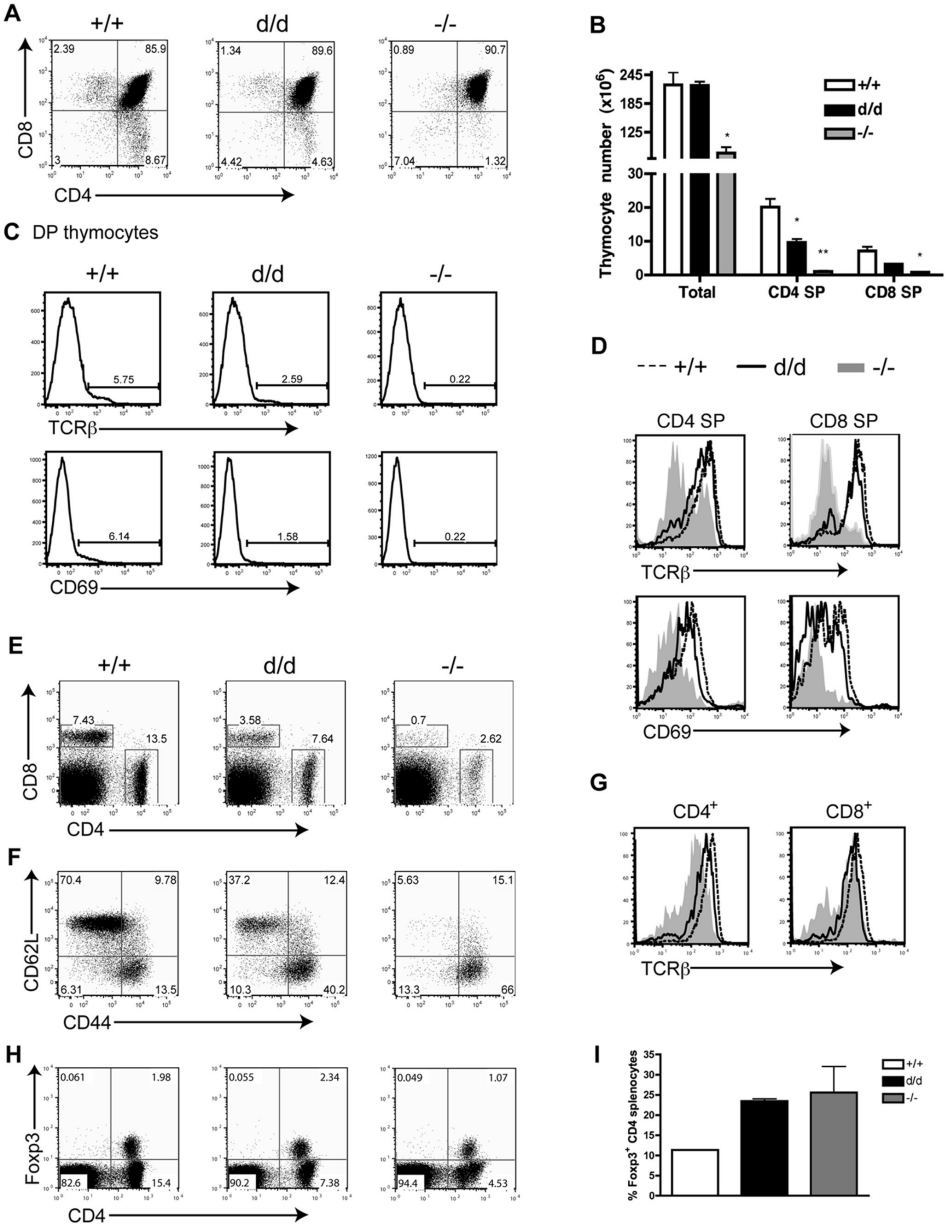
T cell development is impaired in RasGRP1^d/d^ mice. (A) Representative FACS plots of CD8 versus CD4 expression of total live thymocytes from 4- week-old mice. (B) Total numbers of thymocytes, CD4 SP, and CD8 SP populations. (C) Expression of TCRβ (top) and CD69 (bottom) on DP thymocytes. Numbers indicate the percentage of surface marker positive cells. (D) Cell surface expression of TCRβ (top) and CD69 (bottom) on CD4 SP (left) and CD8 SP (right) thymocytes. (E) Representative FACS plot of CD8 versus CD4 on splenocytes from 4-week-old mice. (F) Surface expression of CD44 and CD62L on live, CD4^+^ splenocytes. (G) TCRβ expression on CD4^+^ (left) and CD8^+^ (right) splenocytes. Shaded histogram represents RasGRP1^−/−^, solid black line represents RasGRP1^d/d^, and dashed black line represents RasGRP1^+/+^ thymocytes. (H) Surface expression of CD4 and intracellular expression of Foxp3. (I) The percent of Foxp3^+^ cells in the CD4 splenic compartment was calculated by dividing the percent of Foxp3^+^CD4^+^ cells by the total percent of CD4^+^ cells. Two-tailed *t* test; *, p<0.05; **, p<0.005. Data are representative of at least four independent experiments.

Analysis of 4-week-old RasGRP1^d/d^ mice revealed a defect in thymocyte development. Unlike RasGRP1^−/−^ mice, which had a severe decrease in both SP populations, the percentages of RasGRP1^d/d^ SP thymocytes were only moderately decreased to 1.3% CD8 SP, compared to 2.4% in wildtype mice, and 4.6% CD4 SP, relative to 8.7% in wildtype thymuses (Figure 2A). Whereas total thymic cellularity was not affected in RasGRP1^d/d^ mice, the numbers of CD4 and CD8 SP thymocytes were significantly reduced (Figure 2B). In RasGRP1^−/−^ mice, total thymocyte numbers, along with both SP compartments, were appreciably decreased. Correspondingly, upregulation of TCRβ and CD69, both known indicators of positive selection, was impaired in RasGRP1^d/d^ and RasGRP1^−/−^ DP thymocytes (Figure 2C). While ∼6% of wildtype DP thymocytes upregulated TCRβ and/or CD69, that percentage was reduced to ∼2.5% and ∼1.5%, respectively, in RasGRP1^d/d^ thymocytes. RasGRP1^−/−^ thymocytes had an even greater reduction, only 0.2% upregulated TCRβ and/or CD69. However, TCRβ and CD69 expression on RasGRP1^d/d^ CD4 and CD8 SP thymocytes was not drastically reduced, as it was on RasGRP1^−/−^ CD4 and CD8 SP cells (Figure 2D).

In the periphery, the percentages of CD4^+^ and CD8^+^ T cells in RasGRP1^d/d^ mice were decreased, although not to the extent seen in RasGRP1-deficient mice (Figure 2E). Interestingly, 40% of RasGRP1^d/d^ CD4^+^ T cells in these young mice had upregulated CD44 and downregulated CD62L, while only 15% of wildtype cells exhibited this activated/memory phenotype (Figure 2F). In contrast, 66% of RasGRP1^−/−^ CD4^+^ T cells were CD62L^lo^CD44^hi^, likely a result of homeostatic proliferation following the entry of T cells into the lymphopenic periphery seen in these mice. Furthermore, the surface expression of TCRβ on CD4^+^ and CD8^+^ RasGRP1^d/d^ T cells was slightly reduced, although higher than that on RasGRP1^−/−^ T cells (Figure 2G). Despite the defect in conventional T cell development, RasGRP1^d/d^ and RasGRP1^−/−^ spleens contained distinct populations of Foxp3^+^CD4^+^ regulatory T cells (Figure 2H). In fact, the percent of Foxp3^+^ cells in the CD4^+^ splenic compartment was increased from about 11% in wildtype mice to 20–25% in RasGRP1^d/d^ and RasGRP1^−/−^ mice (Figure 2I). This finding was in agreement with a previous study which demonstrated normal regulatory T cell development and suppressor function in RasGRP1^−/−^ mice [15]. These data showed that the deletion of the tail domain of RasGRP1 led to impaired T cell development, causing reduced numbers of SP thymocytes and mature conventional T cells.

### Splenomegaly and autoimmunity in aged RasGRP1^d/d^ mice

**Figure 3:**
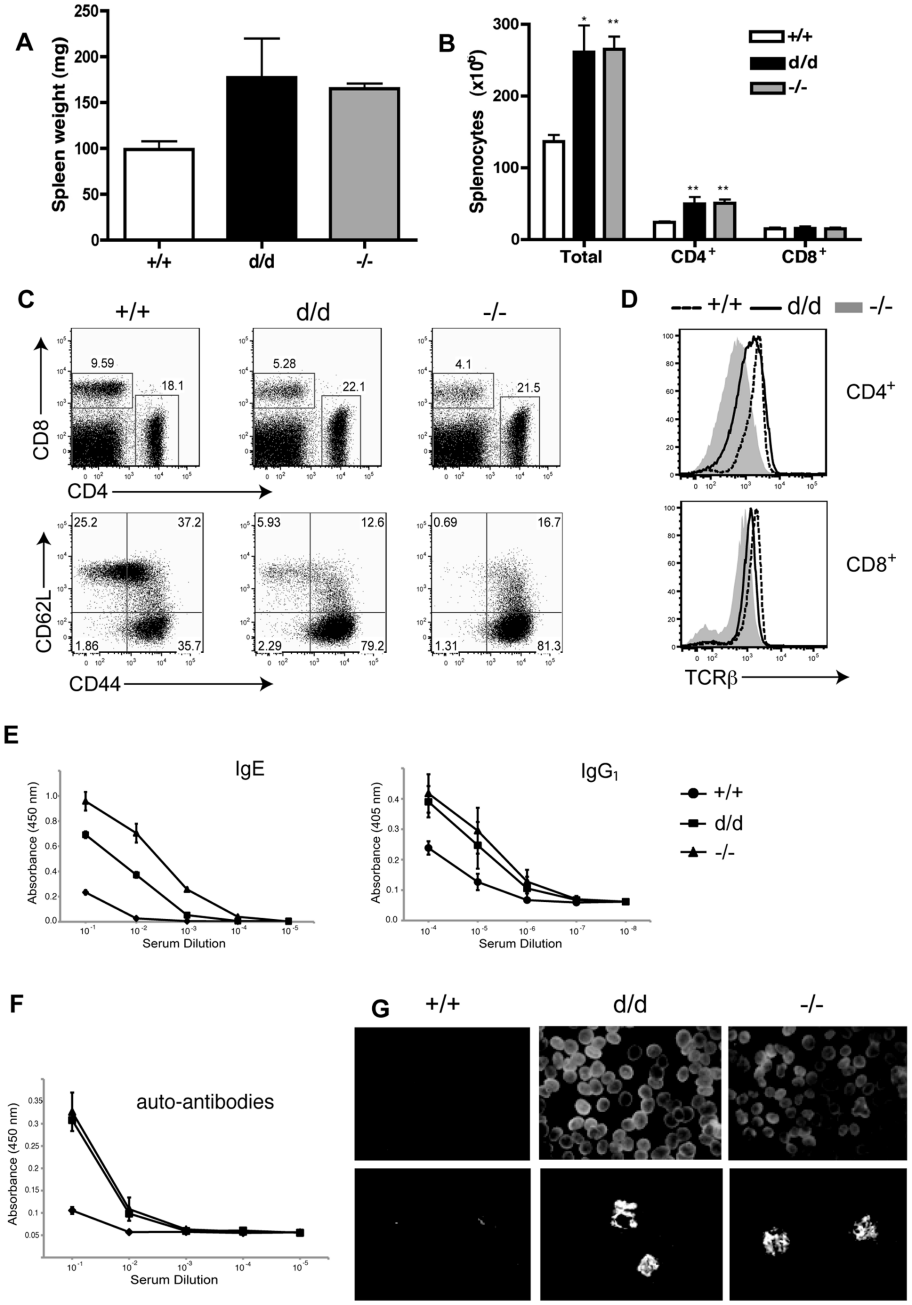
Splenomegaly and autoimmunity in aged RasGRP1^d/d^ mice. (A) Spleen weights from 6-month-old mice. (B) Numbers of total, CD4^+^, and CD8^+^ splenocytes. (C) Representative FACS plots of CD4 versus CD8 on live splenocytes (top), as well as CD62L and CD44 expression on CD4^+^ splenocytes (bottom). (D) TCRβ expression on RasGRP1^−/−^ (shaded histogram), RasGRP1^d/d^ (solid black line), and RasGRP1^+/+^ (dashed black line) splenocytes (left). (E–F) Blood serum levels of IgE, IgG_1_ (E), and anti-nuclear antibodies (F) from RasGRP1^−/−^ (triangles), RasGRP1^d/d^ (squares), and RasGRP1^+/+^ (circles) mice. (G) Top panel, auto-antibodies were detected by immunostaining slides with fixed NIH3T3 cells with mouse serum, followed by FITC-conjugated anti-mouse IgG. Bottom panel, immune complex deposition in the glomeruli of mice was visualized by staining kidney cryosections with FITC-conjugated anti-mouse IgG. Imaging was performed using light or fluorescent microscopy at 10x magnification. Two-tailed *t* test; *, p<0.05; **, p<0.005. Data are representative of at least three independent experiments.

Although RasGRP1^d/d^ mice did not have as severe defects in T cell development as RasGRP1^−/−^ mice, they exhibited splenomegaly at ∼3–4 months of age. At six months of age, spleen weights were greatly increased in RasGRP1^d/d^ and RasGRP1^−/−^ mice, along with overall cell numbers (Figures 3A and 3B). Despite the decreased percentages of CD4^+^ T cells in younger RasGRP1^d/d^ and RasGRP1^−/−^ mice, total numbers of CD4^+^ T cells were significantly increased compared to wildtype mice. CD4^+^ T cells preferentially expanded over CD8^+^ splenocytes, which were comparable in total numbers but decreased in percentages compared to wildtype splenocytes. In wildtype mice, ∼35% of CD4^+^ cells were CD62L^lo^CD44^hi^, whereas ∼80% of RasGRP1^d/d^ and RasGRP1^−/−^ CD4^+^ T cells were CD62L^lo^CD44^hi^ (Figure 3C). Surface expression of TCRβ was also reduced on RasGRP1^d/d^ T cells and further reduced on RasGRP1^−/−^ T cells (Figure 3D).

To evaluate systemic irregularities in these mice, we assayed their serum antibody levels. Although the percentages and activation status of B cells were similar (data not shown), serum from RasGRP1^d/d^ and RasGRP1^−/−^ mice contained higher levels of IgE and IgG_1_, while other isotypes measured, including IgG_2a_, IgG_2b_, and IgM, were comparable (Figure 3E and data not shown). We also performed auto-antibody ELISAs using NIH3T3-coated plates to determine if the RasGRP1 mutation caused an autoimmune-like disorder. As shown in Figure 3F, serum from RasGRP1^d/d^ and RasGRP1^−/−^ mice contained a higher amount of auto-antibodies than that from wildtype mice. Furthermore, staining NIH3T3 cells with serum from RasGRP1^d/d^ and RasGRP1^−/−^ mice revealed striking nuclear staining (Figure 3G, top panel). We also detected immune complexes in the kidneys of RasGRP1^d/d^ and RasGRP1^−/−^ mice (Figure 3G, bottom panel). These data indicated that T cell tolerance and/or homeostasis were disrupted upon the loss of the RasGRP1 tail domain, culminating in auto-antibody production and immune complex deposition.

### Impaired function in RasGRP1^d/d^ T cells

**Figure 4:**
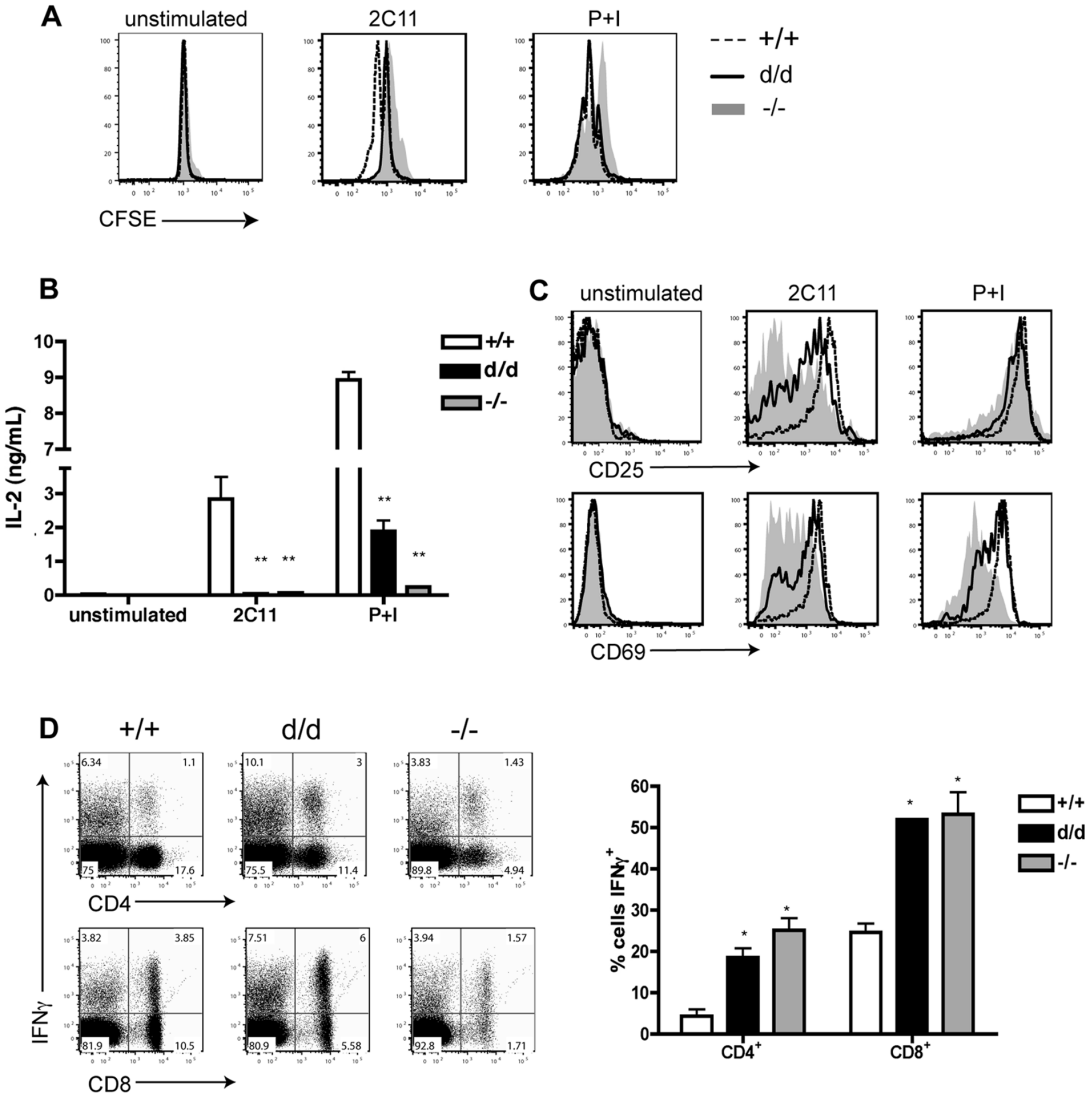
Aberrant T cell proliferation, activation, and cytokine production in RasGRP1^d/d^ mice. (A) Splenocytes were isolated from six to eight-week-old mice and stained with 5 µM CFSE and stimulated as shown. After forty-eight hours, cells were harvested and stained for CD4 and CD8. Histograms shown are gated on CD4^+^ cells. Shaded histogram represents RasGRP1^−/−^, solid black line represents RasGRP1^d/d^, and dashed black line represents RasGRP1^+/+^ splenocytes. (B) Supernatant from the CFSE culture was collected after 24 hours and used to perform an IL-2 ELISA. (C) Cells were stimulated as in (A) but harvested after twenty-four hours and analyzed by FACS for CD4, CD8, CD69, and CD25 expression. Shaded histogram represents RasGRP1^−/−^, solid black line represents RasGRP1^d/d^, and dashed black line represents RasGRP1^+/+^ CD4^+^ splenocytes. (D)Splenocytes from six-week-old mice were harvested and stimulated with P+I with Monensin for 4 hours. Left: Representative FACS plots of splenocytes stained with 7AAD, CD4, CD8, and IFN-γ. Right: Quantitative representation of the percent of CD4 and CD8 cells from each genotype that made IFN-γ following *in vitro* P+I stimulation. Two-tailed *t* test; *, p<0.05; **, p<0.005. Data are representative of at least three independent experiments.

To determine the role of the RasGRP1 tail domain in T cell activation, we assayed the ability of RasGRP1^d/d^ splenic T cells to respond to stimulation. To assess proliferative capacity, we labeled splenocytes with CFSE, stimulated them with PMA and ionomycin (P+I), as well as through the TCR using 2C11, and harvested the cells after 48 hours. CD4^+^ RasGRP1^d/d^ and RasGRP1^−/−^ T cells had slightly impaired proliferation as measured by CFSE dilution in response to 2C11 stimulation (Figure 4A). Upon the addition of P+I, RasGRP1^d/d^ CD4^+^ T cells, but not RasGRP1^−/−^ T cells, were able to proliferate comparably to wildtype cells. ELISA analysis of secreted IL-2 revealed a marked decrease in the presence of IL-2 in RasGRP1^d/d^ and RasGRP1^−/−^ splenocyte cultures, particularly following 2C11 stimulation (Figure 4B). Furthermore, the addition of IL-2 in the presence of 2C11 did not rescue RasGRP1^d/d^ and RasGRP1^−/−^ CD4^+^ proliferation (data not shown).

We next assessed the ability of RasGRP1^d/d^ and RasGRP1^−/−^ CD4^+^ T cells to upregulate activation markers. As seen in Figure 4C, RasGRP1^d/d^ cells increased CD25 expression following 2C11 stimulation, although to a lesser extent than wildtype cells. The upregulation of CD25 on RasGRP1^−/−^ CD4^+^ T cells following 2C11 stimulation was significantly impaired. However, in response to P+I stimulation, CD4^+^ T cells from all three genotypes were able to upregulate CD25. While CD69 upregulation was only slightly defective in RasGRP1^d/d^ CD4^+^ T cells following both types of stimulation, RasGRP1^−/−^ CD4^+^ T cells had a marked defect in their ability to upregulate CD69. CD8^+^ T cells from RasGRP1^d/d^ and RasGRP1^−/−^ mice behaved similarly to CD4^+^ cells but with a less severe defect in their proliferation and activation marker upregulation (data not shown). We further determined the ability of these T cells to produce cytokines by stimulating splenocytes for 4 hours with P+I before intracellular staining. Interestingly, ∼20% of CD4^+^ and over 50% of CD8^+^ T cells from RasGRP1^d/d^ and RasGRP1^−/−^ mice produced IFN-γ. In comparison, ∼5% of CD4^+^ and ∼25% of CD8^+^ T cells from wildtype mice were IFN-γ^+^ (Figure 4D). None of the T cells made a significant amount of IL-4 (data not shown).

### Diminished Erk activation in RasGRP1^d/d^ thymocytes

**Figure 5:**
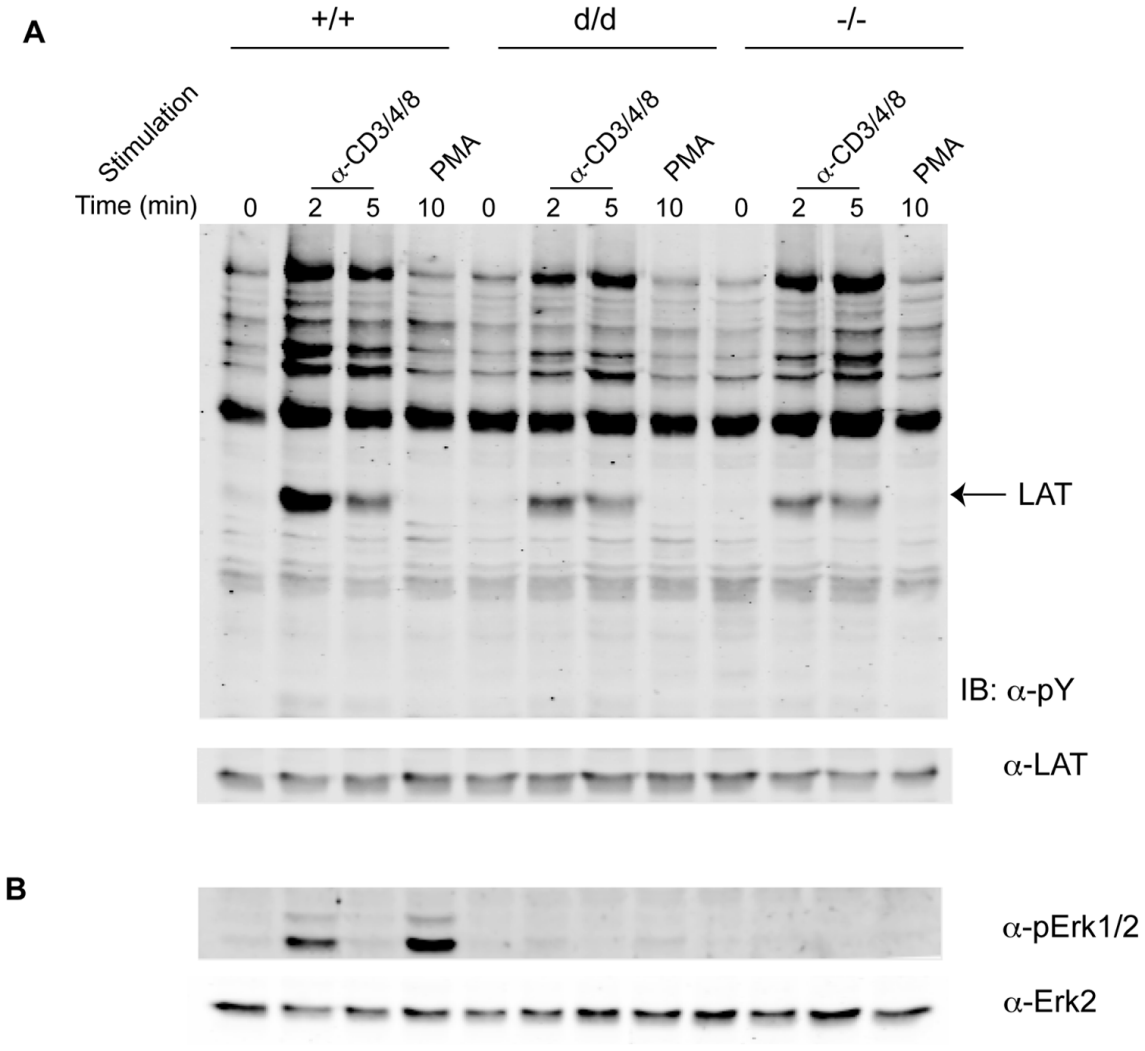
Diminished Erk activation in RasGRP1^d/d^ DP thymocytes. (A–B) Enriched DP thymocytes were stimulated as shown. Lysates were analyzed via SDS-PAGE and immunoblotting for overall tyrosine phosphorylation (pY) and LAT (A) or pErk and Erk2 (B).

As noted in Figure 2C, RasGRP1^d/d^ DP thymocytes did not upregulate TCRβ and CD69 to the extent of wildtype cells, indicating a defect in positive selection. Since Erk activation is essential for positive selection [23], [24], [25], we analyzed Erk phosphorylation in DP thymocytes. Thymocytes were isolated from three-week-old mice and enriched for DP populations to eliminate any differences that might arise from unequal numbers of SP cells. DP thymocytes were then stimulated using anti-CD3/CD4/CD8 antibodies or PMA. Western blots performed on cell lysates revealed similar levels of tyrosine phosphorylation of proteins, with slightly reduced levels of LAT phosphorylation in RasGRP1^d/d^ and RasGRP1^−/−^ thymocytes (Figure 5A). RasGRP1^−/−^ thymocytes could not activate Erk following both types of stimulation (Figure 5B). Unexpectedly, despite having only a slight defect in thymocyte development, RasGRP1^d/d^ DP thymocytes had a severely diminished ability to activate the Erk pathway, signifying major ramifications on positive selection. These data demonstrated that the tail domain of RasGRP1 is critical for TCR-mediated Erk activation.

### Defective thymic selection in RasGRP1^d/d^ mice

**Figure 6:**
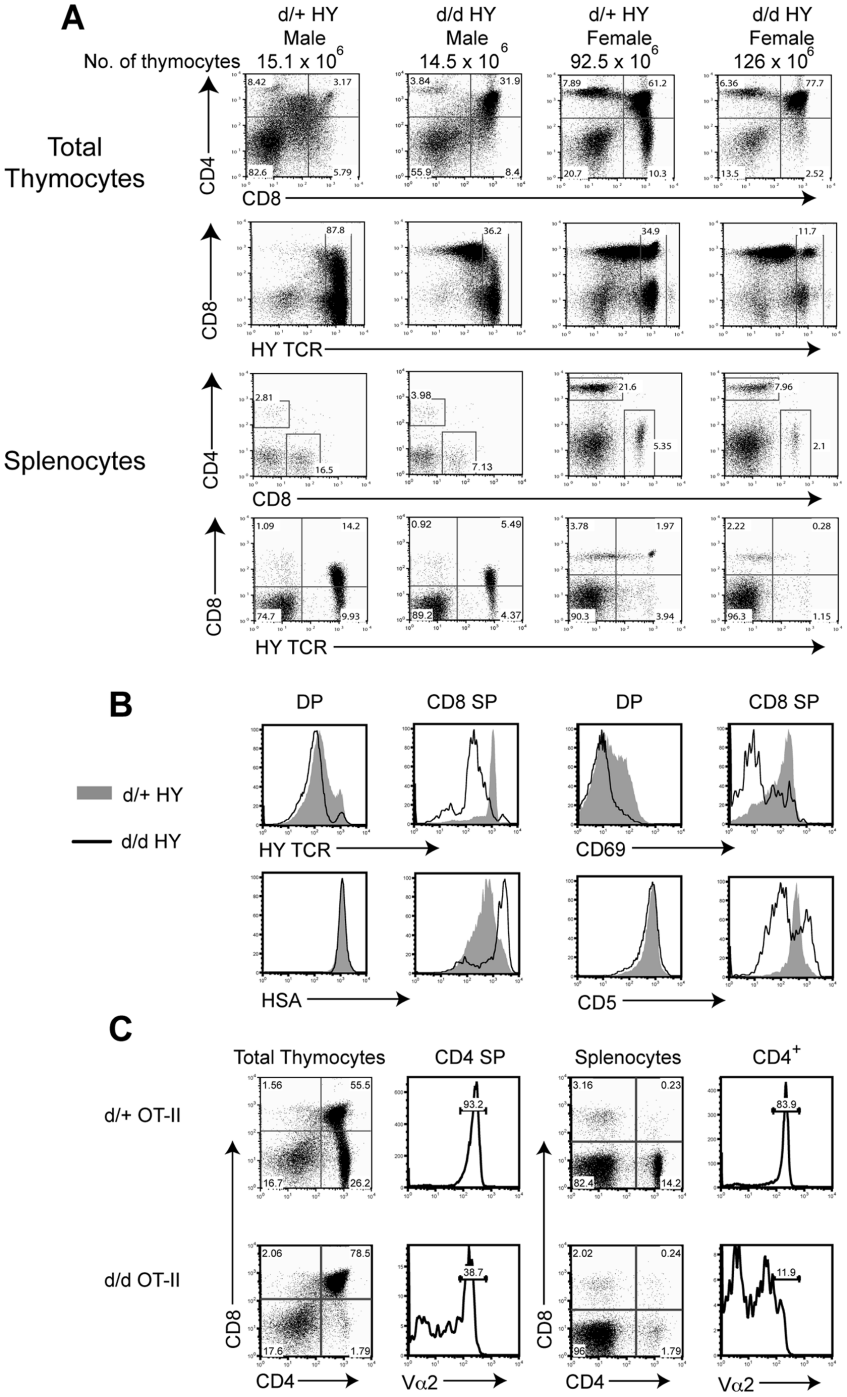
Negative and positive selection defects in RasGRP1^d/d^ mice. (A) Total thymocytes and splenocytes from wildtype and RasGRP1^d/d^ male and female mice expressing the HY TCR transgene were analyzed for the expression of CD4, CD8, and the transgenic TCR chain used by the HY TCR. Numbers above the plots indicate the total number of thymocytes. N=6–9 mice per group. (B) DP and CD8 SP thymocytes from female mice expressing the HY TCR were analyzed for expression of the HY TCR, HSA, CD69, and CD5. The shaded histogram represents wildtype cells while the solid line indicates RasGRP1^d/d^ thymocytes. (C) Thymocytes and splenocytes from wildtype and RasGRP1^d/d^ mice expressing the OT-II TCR were analyzed for the expression of CD4 and CD8. Representative histograms show the levels of Vα2 on either CD4 SP or CD4^+^ splenocytes. The data are representative of four independent experiments.

Considering the impaired thymic development and autoimmune disorder seen in our mice, we decided to look more closely at thymic selection in RasGRP1^d/d^ mice. We bred RasGRP1^d/d^ mice onto the MHC class I-restricted HY TCR transgenic line. RasGRP1^−/−^ mice have similarly been examined on the HY background with the conclusion that RasGRP1 impacts positive, but not negative, selection [12]. The HY system allows for the examination of negative selection in males which express the male-specific HY antigen, thus causing massive deletion of antigen-specific DP thymocytes. Similar numbers of thymocytes were recovered from wildtype HY and RasGRP1^d/d^ HY mice (Figure 6A, top left). However, staining of thymocytes revealed the presence of a significant DP population in RasGRP1^d/d^ HY thymuses that was not seen in wildtype HY mice. While only 3% of thymocytes in wildtype mice were double positive, ∼30% of thymocytes in RasGRP1^d/d^ HY male mice were CD4^+^CD8^+^. However, the CD8 SP compartment was not substantially increased in male RasGRP1^d/d^ mice; there was also no increase in peripheral CD8^+^ T cells in the spleens of these mice. We hypothesized that the lower levels of HY TCR expressed on RasGRP1^d/d^ thymocytes accounted for the increased DP compartment, although this population did not successfully develop into CD8 SP cells.

Upon analysis of RasGRP1^d/d^ HY female mice, we found a marked defect in positive selection. In wildtype HY females, thymocytes expressing the antigen-specific TCR are positively selected, as indicated by an increased percentage of CD8 SP cells. Female RasGRP1^d/d^ HY mice, however, contained far fewer CD8 SP thymocytes, decreased from ∼10% in wildtype to 2.5% in the knock-in mice (Figure 6A, right). Fewer CD8^+^ HY TCR^+^ T cells were also found in the periphery of RasGRP1^d/d^ HY mice. Correspondingly, closer examination of DP thymocytes showed aberrant expression of the HY TCR, CD69, and CD5, while maintaining high expression of HSA (Figure 6B).

In order to assess the requirement of the tail domain during positive selection of MHC class II-restricted TCRs, we crossed RasGRP1^d/d^ mice onto the OT-II TCR transgenic background. Positive selection of OT-II^+^ T cells on an I-A^b^-background results in the accumulation of CD4 SP thymocytes that are Vβ5^hi^Vα2^hi^. Analysis of RasGRP1^d/d^ mice expressing the OT-II-specific TCR revealed a severe defect in the percentage of CD4 SP thymocytes (Figure 6C, left). While 26% of RasGRP1^d/+^ OT-II^+^ thymocytes were CD4 SP cells, RasGRP1^d/d^ OT-II^+^ thymuses contained only ∼2% CD4 SP cells. Furthermore, while over 90% of RasGRP1^d/+^ OT-II^+^ CD4 SP cells stained positive for the Vα2 TCR, only 38.7% of RasGRP1^d/d^ OT-II^+^ CD4 SP cells expressed this TCR. This diminution could be accounted for by either decreased surface TCR expression or the presence of endogenously rearranged TCRs. Likewise, the spleens of RasGRP1^d/d^ OT-II^+^ mice contained far fewer CD4^+^ and Vα2^+^ T cells (Figure 6C, right). Collectively, our data from these two different TCR transgenic lines indicated that the tail domain of RasGRP1 is critical for proper positive and negative selection.

### Impaired localization of RasGRP1Δtail in T cells

**Figure 7:**
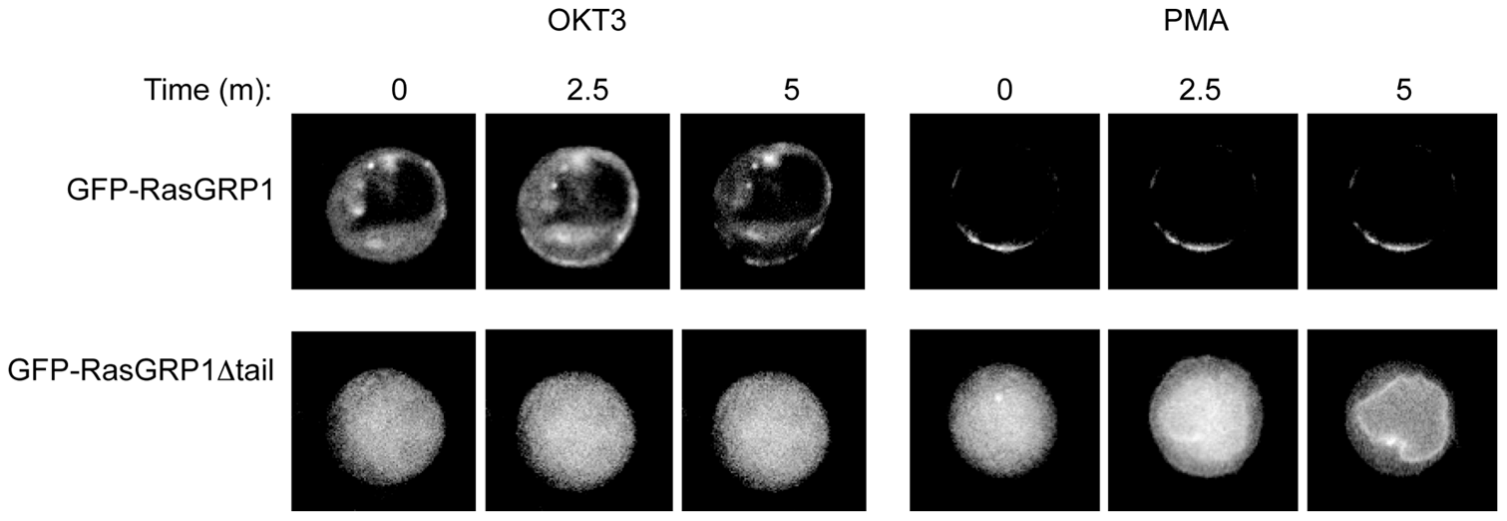
Impaired localization of the RasGRP1Δtail construct in Jurkat T cells. Jurkat cells were electroporated with GFP-RasGRP1 or GFP-RasGRP1Δtail plasmids. Live cell imaging was performed on a Zeiss Observer D1 station equipped with a CoolSNAP_HQ_ charge-coupled device camera (Roper Scientific) and a high-speed automatic objective stage for multiple Z stack recording. The images were collected with a 40x objective lens and 2.5x camera zoom at 45 second intervals at 37°C. For each cell at each time point, GFP data were collected over 21 continuous vertical Z positions. After the first time frame, OKT3 or PMA were added to the samples. Images were processed by 3D deconvolution using AutoQuant X software (Media Cybernetics). All imaging manipulation and analysis were done using the MetaMorph software suite (Molecular Probes). Data are representative of at least six independent experiments analyzing at least five cells per experiment.

In an attempt to elucidate the mechanism by which the tail domain regulates RasGRP1 function and Erk activation, we investigated RasGRP1 translocation following stimulation. Different studies have focused on the localization of RasGRP1 and the ramifications this may have on Ras activation [8], [10], [26], [27], [28]. We wanted to determine if the loss of the tail domain would impact RasGRP1 localization using Jurkat T cells. We electroporated Jurkat cells with GFP-tagged *rasgrp1* wildtype or Δtail construct and performed live cell imaging. The GFP-RasGRP1 wildtype protein was initially found either in the cytoplasm or on the plasma membrane depending upon the level of protein expression (Figure 7). Following stimulation through the TCR via OKT3 or using PMA, GFP fluorescence moved to or intensified at the plasma membrane. In contrast, GFP-RasGRP1Δtail was initially seen throughout the entire cell, including the nucleus. Following OKT3 stimulation, GFP fluorescence failed to move. Interestingly, after the addition of PMA, GFP-RasGRP1Δtail consistently localized in a perinuclear pattern. This aberrant localization was not driven by the GFP tag seeing as similar experiments using Flag-tagged RasGRP1 and RasGRP1Δtail constructs yielded identical results (data not shown). This dramatic defect in protein translocation could account for the inability of the truncated RasGRP1 protein to activate the Ras-Erk pathway.

## Discussion

Recent publications have investigated the roles of the PT (Plasma membrane Targeter) and SuPT (Suppressor of PT) domains, both of which are included in the tail domain. The PT domain was found to be sufficient and necessary for antigen receptor-mediated plasma membrane translocation of RasGRP1 in several B cell lines [10]. A follow-up study defined the precise mechanism by which the PT domain regulates plasma membrane targeting. The authors demonstrate that a small segment of the PT domain, which is enriched in basic/hydrophobic residues, binds directly to PI3K-generated products [11]. In this report, we demonstrated that the tail domain of RasGRP1 was essential for its function in T cell development and activation *in vivo*. The deletion of the tail domain had a significant impact on thymocyte development and T cell function. RasGRP1^d/d^ T cells were intermediary between wildtype and knockout profiles in terms of thymocyte subset percentages and surface marker expression. Despite only a slight defect in T cell development, Erk activation was drastically impaired in RasGRP1^d/d^ DP cells. Furthermore, crossing upon the OT-II and HY transgenic TCR backgrounds led to the near-complete block in positive selection. And, as RasGRP1^d/d^ mice aged, they more closely resembled RasGRP1^−/−^ mice in CD4^+^ T cell percentages, ability of those T cells to produce IFN-γ, and high levels of serum IgE.

In RasGRP1^d/d^ T cells, the remaining RasGRP1 domains may have been able to bypass the need for the tail domain and account for some level of development and activation. The GEF and REM domains are likely required for membrane localization through their binding to Ras, which is anchored in endomembranes or the plasma membrane [29], [30]. Also, the calcium-binding EF hands and the SuPT domain are able to affect plasma membrane targeting of RasGRP1 [10], [29]. Furthermore, the C1 domain mediates both internal membrane and plasma membrane targeting [10], [28]. When the C1 domain is deleted in B cell lines, plasma membrane targeting of RasGRP1 still occurs, albeit with significantly reduced efficiency [10]. In some circumstances, signal strength may be enough to recruit RasGRP1 through its remaining domains, but, with a lower level or different form of signaling, the tail domain may be indispensable. In this manner, we speculate that RasGRP1^d/d^ T cells with a stronger affinity than normal for self-antigen were able to bypass the need for intact RasGRP1 activity and develop into mature T cells.

Additional evidence supporting our claim that the tail domain is essential for RasGRP1 function came from our assessment of T cell function and autoimmunity in RasGRP1^d/d^ mice. RasGRP1^d/d^ T cells exhibited defects in their proliferative capacity, IL-2 production, and upregulation of activation markers, particularly in response to TCR stimulation. Interestingly, despite high levels of serum IgE, T cells from RasGRP1^d/d^ and RasGRP1^−/−^ mice had an increased propensity to produce IFN-γ, but not IL-4, upon stimulation. A report examining TCR-stimulated human cells asserts that naïve CD45RO^−^ T cells stimulated briefly via the TCR produce high levels of IFN-γ, but no IL-4, inducing B cells to generate IgE [31]. Alternatively, as seen in Itk^−/−^ mice [32], γδ T cells could be inducing IgE production by B cells in our mice.

While autoimmunity in RasGRP1^−/−^ mice has been demonstrated previously [13], [14], a conflicting report proposes that those results stem from the mixed background of the mice analyzed [16]. The mice used in the studies described here were backcrossed onto the B6 background for ten generations. By six months of age, the CD4 compartment had expanded significantly and serum from these mice contained high levels of auto-antibodies. From our results, we concluded that the deletion or truncation of RasGRP1 protein impaired thymic selection and/or T cell homeostasis such that these mice indeed developed an autoimmune disorder. The issue of how disrupting RasGRP1 function seems to selectively affect CD4^+^ T cells remains unknown. It has been shown that the CD4^+^ T cell lineage relies more heavily upon Erk signaling [23], [33] and perhaps the loss of RasGRP1 function had more severe ramifications on CD4^+^ rather than CD8^+^ T cells.

Surprisingly, it seems as though peripheral tolerance in these mice was not affected. A previous study documents the functional suppressive nature of RasGRP1^−/−^ regulatory T cells [15]. In accordance with this work, unpublished studies in our lab showed that RasGRP1^−/−^ T cells were able to suppress LATY136F T cell expansion upon co-transfer into lymphopenic mice. Also, the percentage of Foxp3^+^ T cells among the CD4 T cell populations from both RasGRP1^−/−^ and RasGRP1^d/d^ spleens were actually increased compared to wildtype mice (Figure 2I). Regulatory T cells are thought to be selected based on their intermediate affinity for self-ligands in the thymus [34], [35]. The signaling pathways guiding conventional and regulatory T cell development may be intrinsically different and the regulatory T cell lineage may be able to bypass the need for RasGRP1 during its maturation.

Our data clearly showed that deletion of the tail domain affected RasGRP1 protein expression so that it was expressed at about 70% of the level of wildtype protein. A similar effect was seen in human SLE patients [17]. In this study, it was shown that 9 of 13 new splice variants of the human RasGRP1 gene contain a form of RasGRP1 that lacks exons 16 and 17, which comprise most of the tail domain, either alone or in conjunction with other RasGRP1 exons. Moreover, this report shows that the RasGRP1 protein level is reduced in these patients. Thus, our RasGRP1^d/d^ line should be an excellent model to study the human diseases caused by splice variations in the human RasGRP1 gene. While it is possible that the reduced expression of RasGRP1 protein may contribute to the functional defect in RasGRP1^d/d^ mice, RasGRP1^+/−^ cells express a much reduced level of wildtype protein but function normally [2], indicating that the defect observed in RasGRP1^d/d^ mice was a true consequence of the tail domain deletion. Moreover, our data with Jurkat cells clearly showed that deletion of this tail domain impaired RasGRP1 membrane localization. While wildtype GFP-RasGRP1 was concentrated on the plasma membrane, GFP-RasGRP1Δtail was seen throughout the cell and failed to move following TCR engagement. After PMA stimulation, GFP-RasGRP1Δtail moved into a perinuclear pattern, perhaps reflecting the tendency of the C1 domain to accumulate at endomembranes [8], [10], [28]. Thus, our data *in vivo* and *in vitro* implicate an important role for the tail domain in controlling T cell development and autoimmunity. Our study reveals the novel findings that Erk activation in T cells is dependent upon the tail domain of RasGRP1 and that the loss of the RasGRP1 tail domain has severe consequences on thymocyte selection and T cell function.

## Materials and Methods

### Ethics Statement

All experiments with mice were conducted in accordance with National Institutes of Health guidelines. The experiments described here were reviewed and approved by the Duke University Institutional Animal Care Committee (Permit Number: A191-11-07).

### Generation of RasGRP1^d/d^ knock-in mice

RasGRP1 genomic fragments were amplified from embryonic stem (ES) cells by PCR, sequenced, and cloned into the targeting plasmid (Figure 1). The short arm contained a 1.8-kb sequence including exon 14 and a modified exon 15 with a premature stop codon to delete amino acids 669–852. The long arm was comprised of a 4.2-kb sequence including exon 16 and surrounding introns. After transfection, G418-resistant ES cells were screened by PCR. The correctly targeted ES cells were injected into blastocysts to generate chimeric mice. To delete the PGK-Neo cassette, chimeric mice were crossed with β-actin-Cre mice to produce RasGRP1^d/+^ mice. These mice were subsequently backcrossed with C57BL/6 mice for ten generations. Genotyping of littermates was done by PCR using the following primers: 5′-CAT GAA AGC CAT CGG GTA CT-3′ and 5′-AAG ATC CTT CTT CGG GTG CT-3′. To confirm the insertion of a premature stop codon in exon 15, RasGRP1 RNA was amplified by reverse transcription (RT)-PCR using two primers: 5′-TGA CAA CTG TGC TGG CTT TC-3′ and 5′-ACG ATT CTG TTT GGG TGC TC-3′. C57/BL6 mice, OT-II mice, and β-actin-Cre mice were purchased from Jackson Laboratory. HY-TCR transgenic mice were purchased from Taconic Farms. Mice were housed in specific pathogen-free conditions.

### Western blot and real-time PCR analysis

To confirm equal expression of RasGRP1 mRNA, DP thymocytes were sorted and used for total RNA extraction using Trizol reagents. cDNAs were synthesized using SuperScript reverse transcriptase (Invitrogen) using oligo-dT as the primer. Quantification of RasGRP1 RNAs was performed by real-time PCR with SYBR Green Super Mix (Bio-Rad) using the following primers: 5′-AGG ATG CCC TGG AAA AGA AT-3′ and 5′-AGC TGG CAT CCA TCT TGA AC-3′. For Western blot analyses, 5×10^7^ thymocytes or splenocytes were lysed in 1 mL of RIPA buffer. The lysates were resolved on SDS-PAGE and probed with a RasGRP1 monoclonal antibody (Santa Cruz) or polyclonal antisera (J32), which is directed against the catalytic domain of rat RasGRP1 [4]. We calculated wildtype and mutant RasGRP1 band volumes as a ratio of Erk2 expression using Gausssian deconvolution through the Phoretix 1D gel analysis software (TotalLab Limited).

### FACS analysis and antibodies

Fluorescent antibodies used in flow cytometry, such an anti-CD4, -CD8, -CD25, -CD44, -CD62L, -HY TCR, -TCRβ s-HSA, and -Vα2, were all purchased from eBioscience or BioLegend. For surface marker staining, single-cell suspensions were prepared from thymuses or spleens and were blocked with 2.4G2 (anti-FcγII/III receptor) before staining with different antibody mixtures. For intracellular Foxp3 staining, splenocytes were prepared and stained for surface markers as above. They were then fixed and permeabilized with the eBioscience Foxp3 staining kit before being stained with anti-Foxp3 (eBioscience). For intracellular staining of cytokines, cells were isolated and stimulated with PMA (20 ng/mL) and ionomycin (0.5 µg/mL) for one hour before the addition of Monensin. Cells were left for three additional hours before being fixed and permeabilized per the manufacturer's instructions (eBioscience). Cells were then stained with anti-IFN-γ and -IL-4. Flow cytometry data were acquired on FACSCanto (BD Biosciences) and analyzed with the FlowJo software.

### Cell activation, proliferation, and IL-2 production

Splenocytes were stimulated with plate-coated anti-CD3 (3 µg/mL 2C11) or with PMA (20 ng/mL) and ionomycin (0.5 µg/mL) overnight to assess upregulation of activation markers. Cells were then stained with antibodies against CD4, CD8, CD25, CD69, and 7AAD and analyzed via flow cytometry. To determine cell proliferation, splenocytes were loaded with 5 µM CFSE and stimulated with 2C11 or PMA and ionomycin as above. After forty-eight hours, cells were harvested, stained, and analyzed by flow cytometry for CFSE dilution. For IL-2 production, at twenty-four hours after stimulation, 50 µL of supernatant was collected and analyzed by ELISA using IL-2 capture and detection antibodies according to the manufacturer's instructions (eBioscience).

### Serum immunoglobulin and autoimmunity analysis

For serum antibody detection, sera were serially diluted as shown. 96-well plates were coated with anti-mouse IgE and Ig(H+L) and incubated with serum, followed by detection antibodies. For autoantibody detection, 96-well plates were coated with NIH3T3 cells and incubated with serum followed by anti-mouse IgG-HRP. Absorbance at 405 nm (anti-IgE and autoantibody) or 450 nm (all other isotypes) was determined using a precision microplate reader (Molecular Devices). For anti-nuclear antibody fluorescent staining, NIH3T3 cells were blocked with 2.4G2 and then incubated with serum (1∶50) followed by FITC goat anti-mouse IgG. Immune complex deposition in mice was detected by staining cryosections of kidneys with FITC-conjugated goat anti-mouse IgG. Slides were visualized by fluorescent microscopy.

### DP thymocyte isolation, activation, and Western blot analysis

DP thymocytes were enriched using the EasySep biotin selection kit (StemCell Technologies). Briefly, thymocytes were blocked using anti-mouse FcR antibody and then incubated with biotin-conjugated H2-D^b^ antibody (BioLegend). Cells were mixed with biotin selection cocktail and magnetic beads, per the manufacturer's instructions, to remove SP and DN cells which express high levels of MHC class I. Selected thymocytes were then stimulated directly with 20 ng/mL PMA or were incubated with 10 µg/mL biotin-αCD3 and 2.5 µg/mL biotin-αCD4/CD8 and activated by cross-linking with 25 µg/mL streptavidin (Sigma). The cells were lysed at the indicated time points in RIPA lysis buffer. Cell lysates were resolved using SDS-PAGE and transferred onto nitrocellulose membranes (BioRad). Membranes were then blotted with anti-pTyr (Upstate Biotechnology), anti-LAT, anti-pErk (Cell Signaling Technology), and anti-Erk2 (Santa Cruz Biotechnology). For secondary antibodies, AlexaFluor 680 anti-mouse or anti-rabbit IgG (Molecular Probes) or IRDye 800 anti-rabbit (Rockland) were used. The membranes were scanned using the LI-COR Odyssey infrared imaging system.

### GFP-RasGRP1 imaging

The cDNA sequences of mouse *rasgrp1* and *rasgrp1*Δ*tail* (lacking amino acids 665–852) fused to GFP were cloned into the pHSIB retroviral vector. Jurkat cells were electroporated with 10 µg of plasmid DNA. Live cell imaging was performed on a Zeiss Observer D1 station equipped with a CoolSNAP_HQ_ charge-coupled device camera (Roper Scientific) and a high-speed automatic objective stage. Images were collected with a 40x objective lens and 2.5x camera zoom at 45 second intervals. For each cell at each time point, GFP data were collected over 21 continuous vertical Z positions. After 45 seconds, OKT3 or PMA were added to the samples. Images were processed by 3D deconvolution using AutoQuant X software (Media Cybernetics). Imaging manipulation and analyses were done using the MetaMorph software suite (Molecular Probes).
